# Inhibitory Effect of Phenolic Extract from Garlic on Angiotensin-1 Converting Enzyme and Cisplatin induced Lipid Peroxidation – *In Vitro*

**Published:** 2013-06

**Authors:** Ganiyu Oboh, Ayodele J. Akinyemi, Adedayo O. Ademiluyi

**Affiliations:** 1 Functional Foods and Nutraceuticals Unit, Department of Biochemistry, Federal University of Technology, Akure, Nigeria, P.M.B. 704, Akure 340001, Nigeria;; 2 Department of Biochemistry, Afe Babalola University, Ado-Ekiti, Nigeria, P.M.B. 5454, Nigeria

**Keywords:** *Allium sativum*, angiotensin 1 converting enzyme, cisplatin, malondialdehyde, nephroprotective

## Abstract

*Allium sativum* have been an important food ingredient in the management or treatment of renal disease. Therefore, this study sought to determine the inhibitory effect of phenolic-rich extract from *A. sativum* on angiotensin 1 converting enzyme (ACE) activity (key enzyme linked to renal dysfunction) and cisplatin-induced lipid peroxidation in rat kidney *in vitro*. The free phenolics were extracted with 80% acetone, while the bound phenolics were extracted from the alkaline and acid hydrolyzed residue with ethyl acetate. Thereafter, their inhibitory effect on angiotensin 1 converting enzyme (ACE) activity and cisplatin-induced lipid peroxidation in rat kidney were determined-*in vitro*. The results revealed that the free phenolics had significantly higher (*P*<0.05) inhibitory effect on ACE activity than the bound phenolics. Furthermore, incubation of rat kidney in presence of 1 mM cisplatin caused a significant increase (*P*<0.05) in the malondialdehyde (MDA) content, however, both extracts inhibited MDA produced in a dose dependent manner. The additive and/or synergistic action of the free and bound phenolics could have contributed to the observed medicinal properties of the spice. Therefore, inhibition of ACE activity and prevention of oxidative stress in the kidney could be some of the possible mechanism by which they exert nephroprotective properties. However, the bound phenolic extracts showed stronger inhibition on ACE activity *in vitro*.

## INTRODUCTION

Oxidative stress has been implicated to play a role in the progression of renal disease indirectly by promoting hypertension and atherosclerosis or directly by inducing glomerular damage and renal ischemia (1). It results from either a decrease of natural cell antioxidant capacity or an increased amount of reactive oxygen species (ROS) in organisms. Malondialdehyde is the end-product of lipid peroxidation, which is a process where reactive oxygen species degrade polyunsaturated lipids. This compound is a reactive aldehyde and is one of the many reactive electrophile species that cause toxic stress in cells and form advanced glycation end-products. The production of this aldehyde is used as a biomarker to measure the level of oxidative stress in an organism (2).

Cisplatin [cis-diammine dichloride platinum (II) (CDDP)] a platinum-based chemotherapy drug used to treat various types of cancers, including sarcomas, some carcinomas (e.g. small cell lung cancer, and ovarian cancer), lymphomas and germ cell tumors (3). Like other anticancer agents that cause toxicity in various organs by disturbing the oxidant/antioxidant balance (4); cisplatin-induced nephropathy is closely associated with an increase in lipid peroxidation (5). Nephropathy is a condition that affects the function of the kidneys, and that may progress over time to kidney failure. Patients with kidney failure undergo either painful dialysis or kidney transplantation from a willing living donor (6), which is both costly and harmful. Presently, there is an urgent need for new therapeutic modalities for halting progression of kidney disease. Therapy for oxidative stress of the kidney has focused on antioxidants and agents that modify the renin-angiotensin system. Therefore, agents that inhibit angiotensin I-converting enzyme (ACE) has been suggested to be a useful approach for the management/prevention of kidney dysfunction and dietary phytochemicals have promising potential (7).

Spice such as garlic is of major economic and dietary importance all over the world. Garlic (*A. sativum*) has a very long history of use as food ingredients and medicine and they are grown, traded and consumed in most countries. Their bulbs and corms (raw or cooked) are wonderfully nutritious and therapeutic (8). *Allium sativum* is a rich source of organosulfur compounds and volatile oils, which are responsible for its flavor and aroma, as well as for its potential health benefits (8). Investigations conducted on garlic show that the plant has wide and diverse biological activities including antidiabetic, antiatherosclerotic, antithrombotic, antihypertensive, antihyperlipidemic, antiinflammatory, antioxidant, etc (9, 10). In Ayurvedic medicine, spices such as *Allium* species are used for the management/prevention of several ailments such as kidney disease and urinary disorders. However, there is little or no information on the possible mechanism of action by which they exert this effect. Hence, this study seeks to investigate the inhibitory effect of phenolic-rich extracts from garlic (*A. sativum*) on angiotensin I-converting enzyme (key enzyme linked with renal dysfunction) and cisplatin induced lipid peroxidation in rat kidney *in vitro*.

## MATERIALS AND METHODS

### Materials

Fresh sample of garlic (*Allium sativum*) was purchased at the Erekesan market in Akure metropolis, Nigeria. Authentication of the samples was carried out at the Department of Biology, Federal University of Technology, Akure, Nigeria. The samples were air-dried and grinded into fine powder. All chemicals and reagents used in this study were of analytical grade and glass-distilled water was used. A JENWAY UV-visible spectrophotometer (Model 6305; Jenway, Barlo world Scientific, Dunmow, United Kingdom) was used to measure absorbance.

### Extraction of free soluble phenolics

The extraction of free and bound phenolics was carried out according to the method reported by Chu *et al*. (11). 10 g of the ground bulbs was extracted with 80% acetone and was filtered (whatman no. 2) under vacuum. The filtrate was then evaporated using a rotary evaporator under vacuum at 45°C until about 90% of the filtrate had been evaporated. The phenolic extracts were frozen, while the residues were kept for the extraction of bound phenolics.

### Extraction of bound phenolics

The residue from free soluble extraction above was flushed with nitrogen and hydrolyzed with about 20 mL of 4 M NaOH solution at room temperature for 1 hr with shaking. Then, the pH of the mixture adjusted to pH 2 with concentrated HCl and the bound phytochemicals were extracted with ethyl acetate (6 times). The ethyl acetate fractions were then evaporated at 45°C (11). The yield of free and bound phenolic extract from 10 g of ground bulbs are 4.06 g and 1.72 g respectively.

### Methods

**Angiotensin I converting enzyme (ACE) inhibition assay.** ACE inhibition was assayed by a Spectrophotometric method of Cushman and Cheung (12). The substrate [Hippuryl-histidyl-leucine (Bz-Gly-His-Leu)] and ACE from rabbit lung (EC 3.4.15.1) were purchased from Sigma. The amount of cleaved hippuric acid from hippuryl-histidyl-leucine is measured by the enzymatic method. Appropriate dilutions of the extracts (0–200 μL) and 50 μL ACE (EC 3.4.15.1) solution (4 mU/mL) were incubated at 37°C for 15 min. After preincubation, the enzymatic reaction was initiated by adding 150 μL of 8.33 mM of hippuryl-histidylleucine (Bz-Gly-His-Leu) in 125 mM Tris–HCl buffer (pH 8.3) to the mixture and incubated at 37°C for 30 min. After incubation, the reaction was arrested by adding 250 μL of 1 M HCl. The Gly–His bond was then cleaved and the hippuric acid produced by the reaction was extracted with 1.5 mL ethyl acetate. Thereafter the mixture was centrifuged to separate the ethyl acetate layer; then 1 mL of the ethyl acetate layer was transferred to a clean test tube and evaporated. The residue was redissolved in distilled water and its absorbance was measured at 228 nm. The control experiment was performed without the test sample and the ACE inhibitory activity was expressed as percentage inhibition.

$$\%\;\text{Inhibition}\;=\;[({\text{Abs}}_{\text{Control}}\;-\;{\text{Abs}}_{\text{Samples}})/{\text{Abs}}_{\text{Control}}]\;\times \;100$$

**Lipid peroxidation assay.**

**Experimental animals.** Ten male Wistar albino rats weighing 190–250 g were purchased from the Central Animal House, Department of Biochemistry, University of Ilorin, Ilorin, Nigeria. They were housed in stainless steel cages under controlled conditions with a 12-hour/12-hour light/dark cycle, 50% humidity, and temperature of 28°C. The rats were allowed ad libitum access to food and water. The animals were handled in accordance with the procedure approved by the Animal Ethics Committee of the Federal University of Technology, Akure, Nigeria.

**Preparation of Tissue Homogenates.** The rats were decapitated under mild diethyl ether anaesthesia and the kidney was rapidly isolated and placed on ice and weighed. These tissues were subsequently homogenized in cold saline (1/10 w/v) with about 10-up-and –down strokes at approximately 1200 rev/min in a Teflon glass homogenizer. The homogenates were centrifuged for 10 min at 3000× g to yield a pellet that were discarded, and a low-speed supernatant (S1) were kept for lipid peroxidation assay (13).

**Lipid Peroxidation and Thiobarbibutric Acid Reactions.** The lipid peroxidation assay was carried out using the modified method of Ohkawa *et al.* (14), briefly 100 μL S1 fraction was mixed with a reaction mixture containing 30 μL of 0.1 M pH 7.4 Tris-HCl buffer, extract (0–100 μL) and 30 μL of 1 mM cisplatin (0–100 μL). The volume was made up to 300 μL by water before incubation at 37°C for 1hr. The color reaction was developed by adding 300 μL 8.1% SDS (Sodium dodecyl sulphate) to the reaction mixture containing S1, this was subsequently followed by the addition of 500 μL of acetic acid/HCl (pH 3.4) mixture and 500 μL 0.8% TBA (Thiobarbituric acid). This mixture was incubated at 100°C for 1hr. TBARS (Thiobarbituric acid reactive species) produced were measured at 532 nm and the absorbance was compared with that of standard curve using MDA (Malondialdehyde).

Inhibition of cisplatin /H_2_O_2_-induced OH* production. The ability of the extracts to inhibit cisplatin/H_2_O_2_ induced decomposition of deoxyribose was carried out using the modified method of Halliwell and Gutteridge (15). Briefly, the spice (0–100 μL) was added to a reaction mixture containing 120 μL 20 mM deoxyribose, 400 μL 0.1 M phosphate buffer, 40 μL 20 mM hydrogen peroxide and 40 μL 1 mM Cisplatin, and the volume was made to 800 μL with distilled water. The reaction mixture was incubated at 37°C for 30 min, and the reaction was stop by the addition of 0.5 mL of 2.8% TCA (Trichloroacetic acid), this was followed by the addition of 0.4 mL of 0.6% TBA (Thiobarbituric acid) solution. The tubes were subsequently incubated in boiling water for 20 min. The absorbance was measured at 532 nm in spectrophotometer.

**Fe^2+^ chelation assay.** The Fe^2+^ chelating ability of the extracts were determined using a modified method of Minotti and Aust (16) with a slight modification by Puntel *et al*. (17). Freshly prepared 500 μM FeSO_4_ (150 μL) was added to a reaction mixture containing 168 μL 0.1 M Tris-HCl (pH 7.4), 218 μL saline and the extracts (0–25 μL). The reaction mixture was incubated for 5 min, before the addition of 13 μL 0.25% 1,10-phenanthroline (w/v). The absorbance was measured at 510 nm in the spectrophotometer. The Fe (II) chelating ability was subsequently calculated.

**1,1-diphenyl–2 picrylhydrazyl free radical scavenging ability.** The free radical scavenging ability of the extracts against DPPH (1,1-diphenyl–2 picrylhydrazyl) free radical was evaluated as described by Gyamfi *et al.* (18). Briefly, appropriate dilution of the extracts (1 mL) was mixed with 1 mL, 0.4 mM methanolic solution containing DPPH radicals, the mixture was left in the dark for 30 min and the absorbance was taken at 516 nm in the spectrophotometer. The DPPH free radical scavenging ability was subsequently calculated.

**Determination of reducing property.** The reducing property of the extracts was determined by assessing the ability of the extract to reduce FeCl_3_ solution as described by Oyaizu (19). 2.5 mL aliquot was mixed with 2.5 mL 200 mM sodium phosphate buffer (pH 6.6) and 2.5 mL 1% potassium ferricyanide. The mixture was incubated at 50°C for 20 min. and then 2.5 mL 10% trichloroacetic acid was added. This mixture was centrifuged at 650 rpm for 10 min. 5 mL of the supernatant was mixed with an equal volume of water and 1 mL 0.1% ferric chloride. The absorbance was measured at 700 nm in the spectrophotometer. The ferric reducing antioxidant property was subsequently calculated as ascorbic acid equivalent.

**Determination of total phenol content.** The total phenol content was determined according to the method of Singleton *et al*. (20). Briefly, appropriate dilutions of the extracts were oxidized with 2.5 mL 10% Folin-Ciocalteau’s reagent (v/v) and neutralized by 2.0 mL of 7.5% sodium carbonate. The reaction mixture was incubated for 40 minute at 45°C and the absorbance was measured at 765 nm in the spectrophotometer. The total phenol content was subsequently calculated as gallic acid equivalent.

**Determination of total flavonoid content.** The total flavonoid content was determined using a slightly modified method reported by Meda *et al.* (21), briefly 0.5 mL of appropriately diluted sample was mixed with 0.5 mL methanol, 50 μL 10% AlCl_3_, 50 μL 1 M Potassium acetate and 1.4 mL water, and allowed to incubate at room temperature for 30 min. The absorbance of the reaction mixture was subsequently measured at 415 nm in the spectrophotometer. The total flavonoid content was subsequently calculated using quercetin as standard.

**Determination of IC_50_.** In order to determine the IC_50_ values, the percentage of enzyme inhibition of the phenolic extracts was plotted against extract concentrations in μg/mL. The IC_50_ was then calculated using non-linear regression analysis. IC_50_ is defined as the concentration of phenolic extracts required to inhibit 50% of the enzyme activity.

### Data Analysis

The results of triplicate experiments were pooled and expressed as mean ± standard deviation (STD). One way analysis of variance was used to analyze the results and the least significance difference (LSD) was carried out (22).

## RESULTS

**Figure 1:**
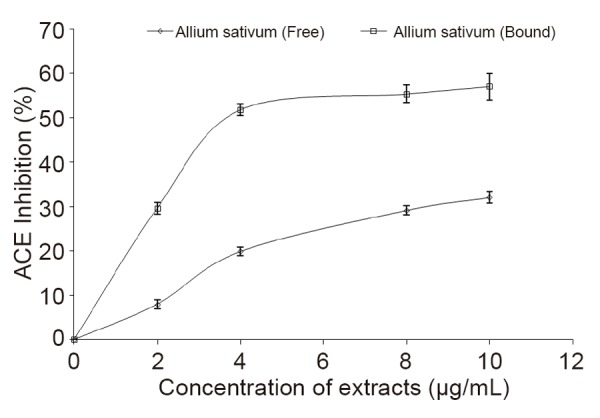
Inhibition of angiotensin 1 converting enzyme (ACE) activity by free and bound phenolic extracts from garlic (*Allium sativum*).

The result of the Inhibition of angiotensin 1 converting enzyme (ACE) activity by free and bound phenolic extracts from garlic (*Allium sativum*) is presented in Figure 1. The result revealed that the phenolic extracts from garlic (*Allium sativum*) inhibited ACE activity in a dose-dependent manner (0–10.0 μg/mL); however, bound phenolics (IC_50_=3.48 μg/mL) had significantly higher (*P*<0.05) inhibitory effect on ACE activity than the free phenolics (IC_50_=14.81 μg/mL).

**Figure 2:**
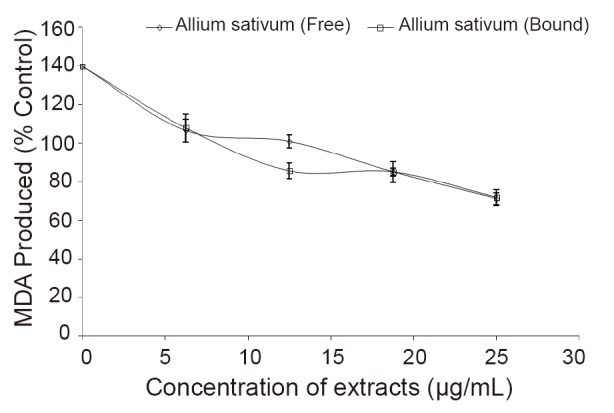
Inhibition of lipid peroxidation in rat kidney by free and bound phenolic extracts from garlic (*Allium sativum*).

Incubating the rat kidney in presence of 1 mM cisplatin caused a significant increase (*P*<0.05) in the malondialdehyde (MDA) content of the tissue homogenate (Figure 2). However, introduction of the free and bound phenolic extracts inhibited MDA production in rat kidney in a dose-dependent manner (0–25 μg/mL), judging by their IC_50_ value (Table 1), there was no significant (*P*<0.05) difference between the free and bound phenolic extracts on the inhibitory effect of cisplatin-induced lipid peroxidation in rat kidney.

**Figure 3:**
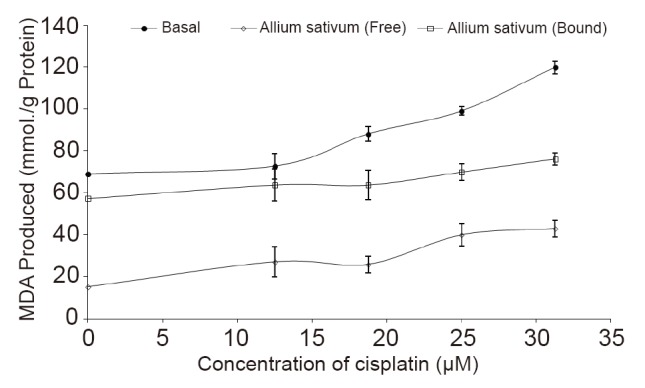
Inhibition of Cisplatin-induced MDA production in rat kidney by free and bound phenolic extracts from garlic (*Allium sativum*) (12.5 μg/mL).

Likewise, incubation of the rat kidney in the presence of cisplatin (0–31.25 μM) caused a dose dependent increase in the MDA content of the kidney (69.08–119.89 mmol./g) as presented in Figure 3. However, introduction of the free and bound phenolic extracts at 12.5 μg/mL inhibited MDA production in rat kidney.

**Figure 4:**
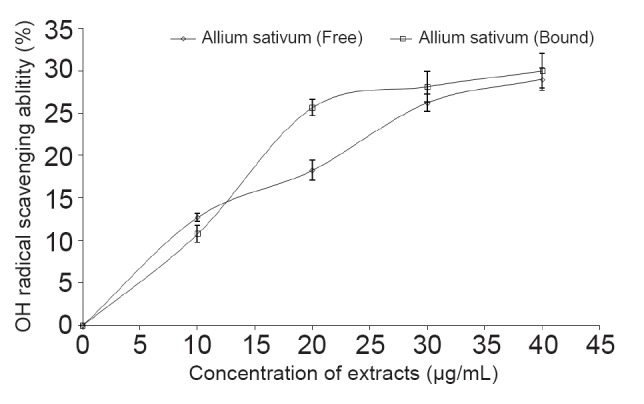
Inhibition of Cisplatin-induced OH* production by free and bound phenolic extracts garlic (*Allium sativum*).

The ability of the phenolic extracts to scavenge OH^•^ produced from the decomposition of deoxyribose in Fenton reaction is presented in Figure 4. The result revealed that the extracts were able to scavenge OH^•^ in a dose-dependent manner (0–40 μg/mL), however judging by their IC_50_ values, there was no significant (*P*<0.05) difference between the free and bound phenolic extracts on OH* scavenging ability.

**Figure 5:**
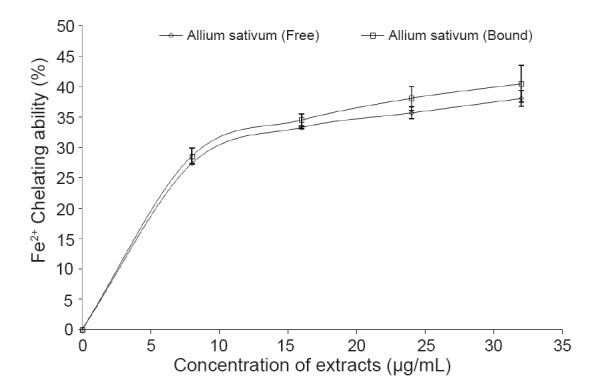
Fe^2+^ chelating ability of free and bound phenolic extracts garlic (*Allium sativum*).

As presented in Figure 5, the phenolic-rich extracts were able to chelate Fe^2+^ in a dose-dependent manner (0–32 μg/mL); nevertheless, there was no significant (*P*<0.05) difference between the free and bound phenolic extracts (Table 1).

**Figure 6:**
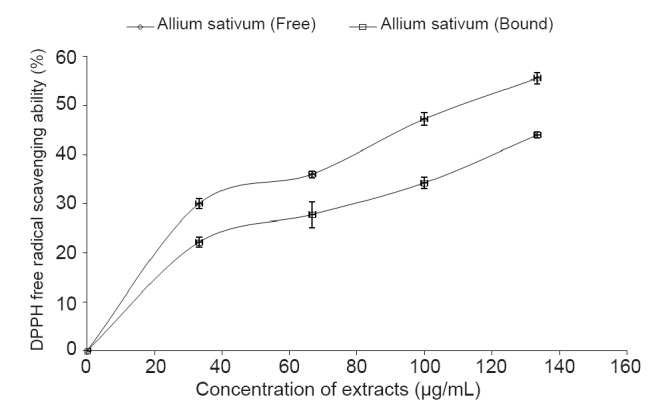
DPPH free radical scavenging ability of free and bound phenolic extracts garlic (*Allium sativum*).

The result as presented in Figure 6 revealed that the extracts scavenged DPPH radicals in a dose-dependent pattern (0–133.33 μg/mL), however judging by their IC_50_ value (Table 1), the free phenolic extracts had significantly higher (*P*<0.05) DPPH radical scavenging ability than the bound phenolic extracts.

**Table 1:** IC_50_ values (μg/mL) of angiotensin 1 converting enzyme (ACE) inhibitory activity, inhibition of lipid peroxidation in rat kidney, Fe^2+^ chelating ability, inhibition of Cisplatin-induced OH* production and DPPH free radical scavenging ability of free and bound phenolic extracts from garlic (*Allium sativum*)

Sample	Free	Bound

ACE inhibitory activity		
Garlic (*Allium sativum*)	10.64^a^ ± 3.63	3.48^b^ ± 2.63
Inhibition of lipid peroxidation		
Garlic (*Allium sativum*)	32.53^a^ ± 0.10	31.47^a^ ± 0.10
Fe^2+^ chelating ability		
Garlic (*Allium sativum*)	11.95^a^ ± 0.30	13.45^a^ ± 0.20
OH* scavenging ability		
Garlic (*Allium sativum*)	8.81^a^ ± 1.26	10.93^a^ ± 21.04
DPPH free radical scavenging ability		
Garlic (*Allium sativum*)	81.51^a^ ± 0.26	125.65^b^ ± 0.40

Values represent means ± standard deviation (n=3). Values with the same superscript along the row are not significantly (*P*<0.05) different.

The reducing powers of the phenolic-rich extracts of *Allium sativum* was assessed based on their ability to reduce Fe^3+^ to Fe^2+^ and the results is presented in Table 2 as ascorbic acid equivalent (AAE). The result revealed that the bound phenolics (133.93 mg. AAE/g) had significantly higher (*P*<0.05) reducing power than the free phenolics (44.64 mg. AAE/g).

**Table 2:** Total phenol content, flavonoid content and ferric reducing antioxidant properties (FRAP) of free and bound phenolic extracts from garlic (*Allium sativum*)

Sample	Free	Bound

Total Phenol (mg. GAE/g)		
Garlic (*Allium sativum*)	32.15^a^ ± 3.63	19.54^b^ ± 2.63
Total Flavonoid (mg. QUE/g)		
Garlic (*Allium sativum*)	21.87^a^ ± 0.18	10.94^b^ ± 0.10
FRAP (mg. AAE/g)		
Garlic (*Allium sativum*)	44.64^a^ ± 1.26	133.93^b^ ± 21.04

Values represent means ± standard deviation (n=3). Values with the same superscript along the row are not significantly (*P*<0.05) different.

The result of the total phenol and flavonoid distribution of *Allium sativum* is presented in Table 2. The result revealed that the free phenolic (32.15 mg. GAE/g) and flavonoid (21.87 mg. QUE/g) content were generally higher than the bound phenolic (19.54 mg. GAE/g) and flavonoid (10.94 mg. QUE/g) content.

## DISCUSSION

Renin-angiotensin system (RAS) plays a pivotal role in blood pressure regulation, salt and water balance, and in the pathophysiology of renal failure and hypertension (23). Renin produces angiotensin I from angiotensinogen, after which it is converted to a potent vasoconstrictor, angiotensin II by angiotensin I converting enzyme (ACE). ACE cleaves angiotensin I to produce angiotensin II, a powerful vasoconstrictor that has been identified as a major factor in renal and hypertension (24). Blockade of the renin angiotensin system (RAS) have a major effect on reducing proteinuria and slowing progression to renal failure (25). Therefore, inhibition of ACE is considered a useful therapeutic approach in the management/treatment of renal dysfunction. This ACE inhibitory property of the phenolic-rich extracts from the *Allium sativum* clearly showed that the extracts could inhibit ACE *in vitro*, and this could explain the possible mechanism for their use in the management/treatment of renal failure in folklore. The inhibition of ACE by the phenolic-rich extracts from the *Allium sativum* agreed with earlier reports on phenolic extracts of bitter leaf (26) and soyabean (27). ACE inhibitors have been widely developed to prevent angiotensin II production in renal dysfunction, and this have been utilized in clinical applications since the discovery of ACE inhibitors in snake venom (28).

Cisplatin, a platinum-based drug used to treat various types of cancers, have been reported to cause nephrotoxicty through their ability to induce lipid peroxidation *in vitro* (29). Therefore, inhibition of cisplatin-induced lipid peroxidation is a useful approach in the prevention of side effect arising from this drug. As presented in Figure 2, free and bound phenolic extracts from garlic (*Allium sativum*) prevented cisplatin-induced lipid peroxidation in rat kidney (*in vitro*). This inhibition in MDA production may be because the extracts are rich in biologically active phenolic compounds with antioxidant activities (30, 31). Nevertheless, the result agrees with an earlier work by Oboh (29), for green and sour tea polyphenols.

In addition, incubation of the rat kidney in the presence of cisplatin (0–119.89 μM) caused a dose dependent increase in the MDA content of the kidney as presented in Figure 3. This increase in MDA content of the kidney confirmed that cisplatin could cause nephrotoxicity by an increase in lipid peroxidation. However, introduction of the free and bound phenolic extracts inhibited MDA production in rat kidney. This high protective effect of the phenolic extracts, clearly points to the possibility that *Allium sativum* could be used in the prevention/management of the renal dysfunction associated with oxidative stress.

Furthermore, the mechanism through which the phenolic-rich extracts prevent cisplatin-induced MDA production were subsequently evaluated by determining their antioxidant properties and the ability of the extracts to inhibit cisplatin-induced hydroxyl radical (OH^•^) production (Figure 4). Cisplatin catalyzed the decomposition of deoxyribose in presence of H_2_O_2_, which indicates that it could induce the production of OH*. However, the results revealed that the phenolic extracts were able to scavenge OH^•^ produced from the decomposition of deoxyribose in Fenton reaction in a dose-dependent manner.

Furthermore, the ability of antioxidants to chelate and deactivate transition metals, prevent such metals from participating in the initiation of lipid peroxidation and oxidative stress through metal catalysed reaction (32). Iron (Fe), an essential metal needed for normal cellular physiology is present in biological systems bound to several protein moieties such as heamoglobin, ferritin etc. It may also exist in free forms in which it is able to participate in Fenton reaction with hydroxyl radical and Fe^3+^ as products. Chelation of such transition metals such as Fe is regarded as a preventive antioxidant mechanism. As presented in Figure 5, the phenolic-rich extracts were able to chelate Fe^2+^ in a dose-dependent manner. This Fe^2+^ chelating ability of the extracts is of immense importance in the protective ability of antioxidant phytochemicals against oxidative stress, as this will make free Fe biologically unavailable thereby inhibiting its role in activating free radicals. However, the Fe^2+^ chelating ability of the phenolic extracts of the spice as presented in Figure 5; could be attributed to the presence of two or more of the following functional groups: -OH, -SH, -COOH, PO_3_H_2_, C=O, -NR_2_, -S- and –O- in a favourable structure–function configuration (33-35).

Antioxidants carry out their protective properties on cells either by preventing the production of free radicals or by neutralizing free radicals produced in the body (36). Prevention of the chain initiation step by scavenging various reactive species such as free radicals is considered to be an important antioxidant mode of action (37); hence, the ability of free and bound phenolics of *Allium sativum* to scavenge free radicals was assessed in this study. This is based on a model system whereby antioxidant capacity is measured by the ability to donate a hydrogen atom thereby neutralizing DPPH radicals. DPPH is a free radical donor that accepts an electron or hydrogen to become a stable diamagnetic molecule (38). The tendencies of electron or hydrogen donation are critical factors in characterizing antioxidant activity that involves free radical scavenging (39).

Reducing power is a novel antioxidation defence mechanism; the two mechanisms available to affect this property are by electron transfer and hydrogen atom transfer (40). This is because the ferric-to-ferrous ion reduction occurs rapidly with all reductants with half reaction reduction potentials above that of Fe^3+^/Fe^2+^, the values in the Ferric reducing antioxidant property (FRAP) assay will express the corresponding concentration of electron-donating antioxidants (41). The result revealed that the bound phenolics had significantly higher (*P*<0.05) reducing power than the free phenolics. Since the antioxidant activity of phenolics is mainly due to their redox properties, this allows them to act as reducing agents, hydrogen donors and singlet oxygen quenchers (42).

Phenolic acids and polyphenolics are among the most abundant antioxidants in plant foods. In addition, to their physiological roles in plants as an important contributor to the survival of plant species they have benefits as antioxidants for human health (43). The result of the total phenol and flavonoid distribution of *Allium sativum* as presented in Table 2 revealed that the free phenolic and flavonoid content were generally higher than the bound phenolic and flavonoid content. The phenolic distribution in *Allium sativum* as shown in Table 2 agrees with the phenolic distribution in many plant foods such as fruits (11), vegetables (44) and peppers (43), because they have more free phenolic than the bound phenolic content. However, the free phenolic content of the *Allium sativum* was significantly higher (*P<*0.05) than the free phenolic content of red pepper, potato, lettuce, cucumber, carrot, spinach, cranberry and broccoli (11, 43, 44); but lower than that of green and sour teas (37). Likewise, the bound phenolics content was higher than that of broccoli, cucumber and red pepper (11, 43, 44).

Phenolics are capable of scavenging free radicals, chelate metal catalysts, activate antioxidant enzymes, reduce α-tocopherol radicals, and inhibit oxidases (45, 46). Their potent antioxidant activity is due to the redox properties of their hydroxyl groups (42, 47). Phenolics are present in plant, in both free and bound forms; bound phenolics mainly in the form of β-glycosides, may survive human stomach and small intestine digestion and reach the colon intact, where they are released and exert their bioactivity (48); while free phenolics are more readily absorbed and thus, exert beneficial bioactivities in early digestion; however, the significance of bound phytochemicals to human health is not clear (11, 44). However, it is possible that different plant foods with different amounts of bound phytochemicals can be digested and absorbed at different sites of the gastrointestinal tract and play their unique health benefits. Bound phytochemicals, mainly in β-glycosides, cannot be digested by human enzymes and could survive stomach and small intestine digestion to reach the colon and be digested by bacteria flora to release phytochemicals locally to have health benefits (11).

## CONCLUSION

In conclusion, *Allium sativum* inhibited angiotensin 1 converting enzyme (ACE) and also protect the kidney from cisplatin-induced lipid peroxidation *in vitro*. However, these properties could be attributed to the additive and/or synergistic action of the free and bound phenolics. Therefore, some possible mechanism by which *Allium sativum* exert renoprotective properties could be through inhibition of angiotensin 1 converting enzyme activity and prevention of lipid peroxidation in the kidney. However, the bound phenolic extracts showed stronger inhibition on ACE activity *in vitro* than the free phenolic extracts.
